# Supplementary material for: The Involvement of Endogenous Enkephalins in Glucose Homeostasis
Source: Biomedicines. 2023 Feb 22;11(3):671. doi: 10.3390/biomedicines11030671 (PMC10045905; doi:10.3390/biomedicines11030671)
Supplement: Supplementary file 1 [file biomedicines-11-00671-s001.zip › biomedicines-2184478-supplementary.pdf]

Supplemental Materials

The role of enkephalins in glucose homeostasis in mice fed a regular diet: To determine the role of enkephalin in glucose homeostasis in mice fed a regular diet, an oral glucose tolerance test (OGTT) was conducted in two different sets (n = 2-4 mice per cohort) of mice (n = 5-7 mice per genotype). The protocol was according to our earlier report [23] except mice were fasted for 6 h (8 AM – 2 PM) with ad libitum access to water during this period. While animals were still kept fasted, a small piece of the tail was snipped, and the baseline blood glucose level was measured for each mouse, using a glucometer (Embrace Omnis Blood Glucose Monitoring System/Omnis Health Embrace Blood Glucose Test Strip). Mice were then weighed and administered a 2 g/kg of glucose solution via oral gavage. Blood glucose levels were then measured at 30, 60, 90, 120 minutes after the glucose gavage. At the end of the experiment, mice were restored with ad libitum access to water and food. Our results showed a comparable glucose tolerance test in mice lacking enkephalins and their wildtype controls (Fig. 1S). Interestingly, the basal levels of glucose was higher in these mice as these mice were older (8-10 month old). Two-way repeated measures ANOVA revealed a significant effect of time (%3,30 = 24.96; P<0.0001) but no effect of genotype ($F_{1,10}$ = 0.23: P=0.63) or interaction between genotype and time ($F_{3,30}$ = 0.27; P = 0.85). This result suggests that neither basal plasma glucose level nor elevated level of plasma glucose level induced by the oral glucose gavage is altered in the absence of enkephalins.

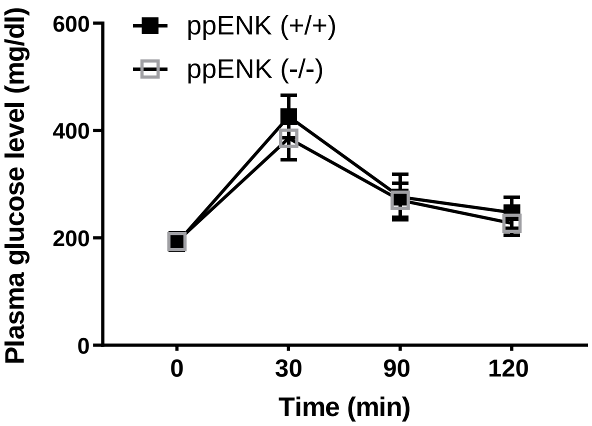

Figure S1. Plasma glucose level was unaltered in the absence of enkephalins in mice fed a regular chow diet. Mice lacking enkephalins and their wildtype littermates/controls were fasted for 6 h, and blood glucose was recorded at the end of this period to measure fasting plasma glucose level. Mice were then challenged with glucose (2 g/kg, p.o.) and blood glucose level was measured again at 30, 60, 90 and 120 min after the oral glucose challenge. Repeated measures ANOVA revealed no significant difference between mice of the two genotypes (P>0.05)
